# The Quantitative Proteome of the Cement and Adhesive Gland of the Pedunculate Barnacle, *Pollicipes pollicipes*

**DOI:** 10.3390/ijms21072524

**Published:** 2020-04-05

**Authors:** Dany Domínguez-Pérez, Daniela Almeida, Josef Wissing, André M. Machado, Lothar Jänsch, Luís Filipe Castro, Agostinho Antunes, Vitor Vasconcelos, Alexandre Campos, Isabel Cunha

**Affiliations:** 1 CIIMAR–Interdisciplinary Centre of Marine and Environmental Research, University of Porto, Rua General Norton de Matos s/n, Terminal de Cruzeiros do Porto de Leixões, 4450-208 Matosinhos, Portugal; dany.perez@ciimar.up.pt (D.D.-P.); danielaalmeida23@gmail.com (D.A.); andre.machado@ciimar.up.pt (A.M.M.); filipe.castro@ciimar.up.pt (L.F.C.); aantunes@ciimar.up.pt (A.A.); vmvascon@fc.up.pt (V.V.); amoclclix@gmail.com (A.C.); 2 Cellular Proteomics Research, Helmholtz Centre for Infection Research, Inhoffenstraße. 7, 38124 Braunschweig, Germany; Josef.Wissing@helmholtz-hzi.de (J.W.); Lothar.Jaensch@Helmholtz-HZI.de (L.J.); 3 Biology Department, Faculty of Sciences, University of Porto, Rua do Campo Alegre, s/n, 4169-007 Porto, Portugal

**Keywords:** underwater adhesion, cement protein, Q-Exactive, proteogenomic, MaxQuant, iBAQ, protein expression

## Abstract

Adhesive secretion has a fundamental role in barnacles’ survival, keeping them in an adequate position on the substrate under a variety of hydrologic regimes. It arouses special interest for industrial applications, such as antifouling strategies, underwater industrial and surgical glues, and dental composites. This study was focused on the goose barnacle *Pollicipes pollicipes* adhesion system, a species that lives in the Eastern Atlantic strongly exposed intertidal rocky shores and cliffs. The protein composition of *P. pollicipes* cement multicomplex and cement gland was quantitatively studied using a label-free LC-MS high-throughput proteomic analysis, searched against a custom transcriptome-derived database. Overall, 11,755 peptide sequences were identified in the gland while 2880 peptide sequences were detected in the cement, clustered in 1616 and 1568 protein groups, respectively. The gland proteome was dominated by proteins of the muscle, cytoskeleton, and some uncharacterized proteins, while the cement was, for the first time, reported to be composed by nearly 50% of proteins that are not canonical cement proteins, mainly unannotated proteins, chemical cues, and protease inhibitors, among others. Bulk adhesive proteins accounted for one-third of the cement proteome, with CP52k being the most abundant. Some unannotated proteins highly expressed in the proteomes, as well as at the transcriptomic level, showed similar physicochemical properties to the known surface-coupling barnacle adhesive proteins while the function of the others remains to be discovered. New quantitative and qualitative clues are provided to understand the diversity and function of proteins in the cement of stalked barnacles, contributing to the whole adhesion model in Cirripedia.

## 1. Introduction

**Figure 1:**
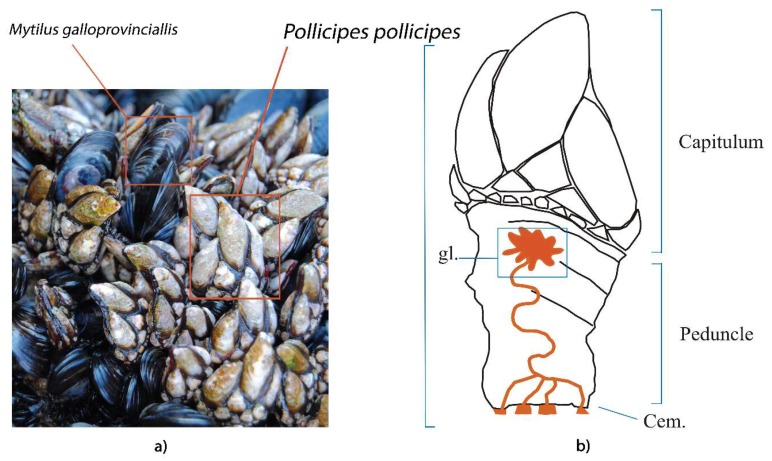
The goose barnacle *Pollicipes pollicipes* (Crustacea: Cirripedia) (**a**) attached to the rocky shore forming conspecific clusters alongside mussels; (**b**) schematic drawing showing sections of the peduncle, highlighting the cement apparatus, formed by clusters of adhesive-secreting cells that together constitute the gland (gl.), located in the upper central core embedded in an apical layer of connective tissue, just beneath the capitulum. Gland secretion passes through a network of ducts that carry the adhesive to the base of the peduncle forming the cement (Cem.).

Sessile marine organisms are bioinspiring for the development of new biomimetic materials and mechanisms. Barnacles’ attachment is very plastic, as they live holdfast to a variety of substrates, under a variety of hydrologic regimes, in oceanic or coastal habitats, submersed or intermittently immersed in intertidal zones, in protected overhangs, crevices, in the deep sea, or directly exposed to strong waves [1,2]. Adhesive secretion has been evolutionarily optimized, since firm and permanent attachment to the substrate is crucial for goose barnacles’ survival, keeping them in an adequate position on the rocks to meet their oxygen and food requirements, grouped in turfs with hard plates facing outside, to protect their soft stalks from predators, and close enough to mate to conspecifics (Figure 1a) since their fecundation is internal [3]. The practical interest in understanding the adhesion of barnacles is related to industrial applications, including the development of antifouling strategies, induction on demand of larvae settlement and fixation for aquaculture, and mimicking the nature-inspired chemistry in new functional materials, such as underwater industrial glues, surgical and dental composites and glues, and biocompatible scaffolds and coatings, which function under humid or wet conditions [4,5,6,7,8].

The original model of adult barnacle’s underwater adhesion [9] describes that the cement is secreted to the space between the animal cuticle and the external substrate, containing mainly two types of proteins, interfacial and bulk proteins. The formers are hydrophilic and located at the periphery of the cement complex, providing surface coupling with exterior surfaces through non-covalent interactions, and also interaction with interior hydrophobic bulk proteins. This model has been updated in the last years [10,11,12], including the notion that surface coupling relies on catechol groups, through various adhesive and cohesive forces, and that catechol, quinone, and lysine-based cross-linking are also involved in protein nanofibers’ curing and proper holdfast to the adhesion surface [10]. However, in barnacles, the catechol groups responsible for β-amyloid fibrils’ cross-linking of bulk proteins do not have a peptidyl origin, differently from other marine organisms, e.g., mussels and sandcastle worms; non-peptidyl catechol precursors and lysine are incorporated among bulk cement protein (CP52k and CP100k) fibrils through enzymatic reactions that involve peroxidases and lysyl oxidase [10], providing structure and mechanical characteristics to the composite. Six barnacle-specific cement proteins (CPs) have been identified, four of which are thought to be interface proteins, CP19k, −20 k, −43 k, and −68 k, and two bulk proteins, CP52k and CP100k, [13]. Barnacle-specific CPs are those proteins present in the cement that share no homology with any other marine adhesive proteins [13,14] or any other proteins at all.

The cement apparatus of the goose barnacle *Pollicipes pollicipes* (Crustacea: Cirripedia) is found at the top of the peduncle core, formed of clusters of a single type of adhesive-secreting unicellular gland, mostly just below the mantle cavity, and a network of ducts that coalesce and carry the adhesive to the base of the peduncle (Figure 1b) [15]. In ripened individuals, some adhesive-secreting cells are intermingled with the ovary, but most of them are between the ovary and the capitulum. The presence of large nucleoli in the nucleus and the large amounts of rough endoplasmic reticulum in the cytoplasm of these cells suggest an intense protein synthesis. The cytoplasm of the adhesive-secreting cells also features numerous small electron-dense secretory vesicles, which stain positively for proteins (tetrazonium) and polysaccharides (PASs) but not for the presence of lipids (Sudan black) in histological studies [15]. Contrarily, on barnacles’ cyprids, the adhesive is reported to be a bi-phasic system containing lipids and phosphoproteins, the two distinct phases contained in two different granule kinds, in the cyprid cement gland cells [16]. Additionally, post-translational modifications do not seem to play a role in adult barnacles’ adhesion, except for the glycosylation of MrCP52k [17], which is in line with the positive PAS dying observed of the electron-dense secretory vesicles in the gland cells’ cytoplasm.

This study focused on the *P. pollicipes* adhesion system (Figure 1), a species that lives in the Easten Atlantic strongly exposed intertidal rocky shores and cliffs, from the south-west British Isles and France to Morocco [18]. The protein composition of *P. pollicipes* cement multicomplex and cement gland was quantitatively studied using label-free LC-MS high-throughput proteomic analysis combined with bioinformatics approaches. The proteogenomic analyses applied allowed the identification of known CPs both in the proteome and gland transcriptome, as well as a group of unknown proteins in the cement proteome. These unknown proteins lacked annotation or conserved domains and were detected as being highly expressed in the cement proteome, gland proteome, and gland transcriptome. In addition, the analysis of some physico-chemical features, such as the molecular weight, isoelectric point, hydrophobicity, amino acid relative composition, secondary structure composition, and disorder of proteins, allowed the conclusion that some of those unannotated proteins could be considered as a new group of surface-coupling cement proteins. We expect that the thorough quantitative description of the proteins found in these two samples, of a pedunculate instead of an acorn barnacle, will provide clues to support recent barnacles’ cement adhesion model updates and help to shed complimentary ideas to design the whole picture of adhesion in Cirripedia.

## 2. Results

### 2.1. Protein Identification

**Figure 2:**
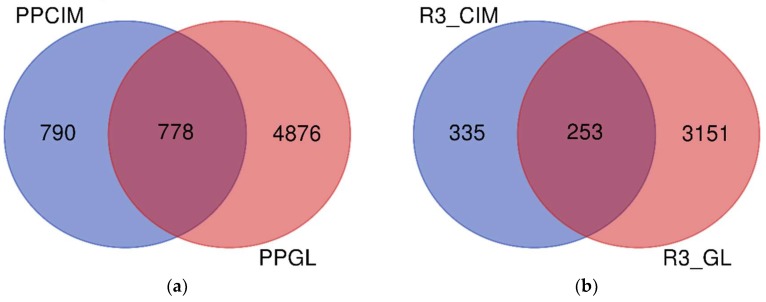
Venn diagram of the proteins identified with MaxQuant freeware in the cement and gland proteome of the barnacle *P. pollicipes*. Unique and shared proteins between (**a**) all proteins identified in the cement (PPCIM) and gland proteome (PPGL); and (**b**) all proteins identified in the three biological replicates of the cement (R3_CIM) and gland proteome (R3_GL).

The shotgun proteomics approach employed to profile the proteome of the cement gland and cement of the stalked barnacle *Pollicipes pollicipes* (Figure 1) allowed the identification of 11,755 peptide sequences in the gland (Table S1) and 2880 peptide sequences in the cement (Table S2). After filtering (contaminants and REV_ removal), a total of 5654 proteins clustered in 1616 proteinGroups remained in the gland proteome (Table S3), and 1568 proteins clustered in 489 proteinGroups in the cement (Table S4). Altogether, 6644 unique proteins were identified, with 778 proteins shared between the gland and cement (Figure 2a). Of all the proteins identified, 3404 and 588 were found in the three replicates of the gland samples and cement, accounting for 889 and 152 proteinGroups, respectively (Table S5 and Table S6). Among them, 3739 were unique proteins whilst 253 were found in all replicates analyzed (Figure 2b). The original MaxQuant output files containing all proteinGroups without filtering can be found in Table S7 for the gland and in Table S8 for the cement.

### 2.2. Quantitative Proteomic Analyses

**Figure 3:**
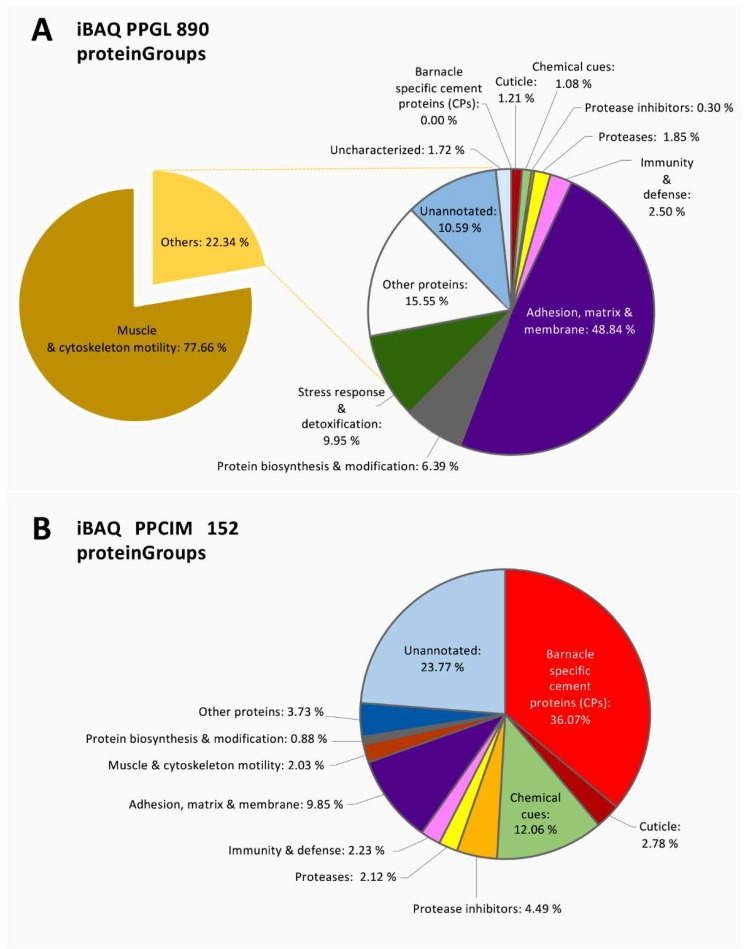
Global protein composition by functional groups in (**a**) the gland proteome (PPGL); and (**b**) cement proteome (PPCIM) of the barnacle *Pollicipes pollicipes*. The proportion of functional groups was based on the absolute protein abundance using the intensity-based absolute quantification (iBAQ) score calculated by MaxQuant. Only those proteins found in the three biological replicates (three valid values) of the two studied samples were selected. In total, 890 proteinGroups were used in the analysis of the gland (PPGL), and 152 in the cement (PPCIM).

**Figure 4:**
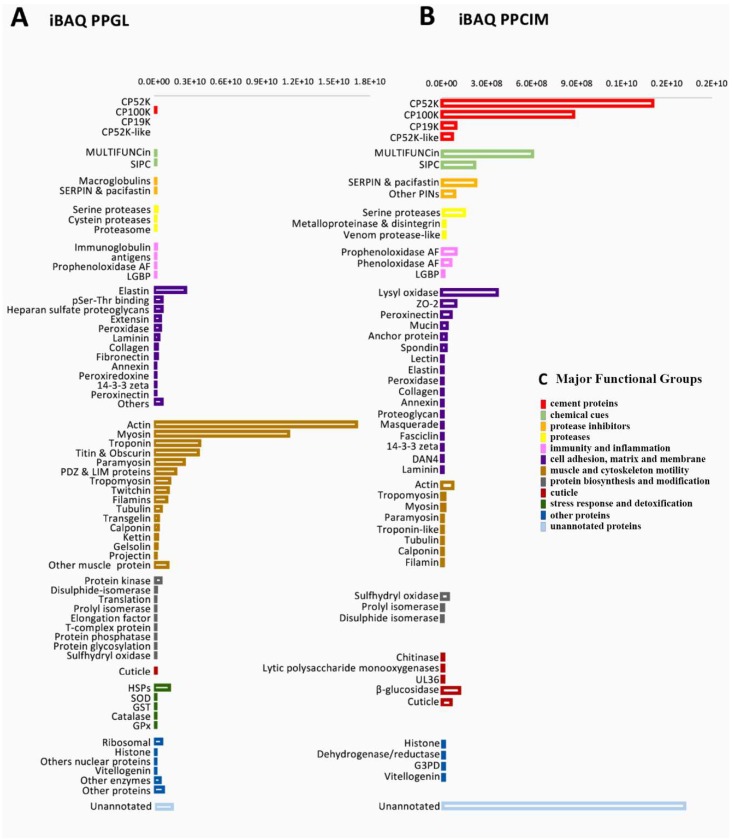
Relative protein abundance within (**a**) the gland proteome (PPGL); and (**b**) cement proteome (PPCIM) of the barnacle *Pollicipes pollicipes*. Absolute protein abundance of broad protein families, shown as a single bar, was obtained using an intensity-based absolute quantification (iBAQ) score calculated by MaxQuant. The horizontal scales of bars differ between samples (PPGL and PPCIM), being the highest in the gland proteome. Major functional groups (**c**) corresponding to the protein families identified are represented by color bars on the right side.

Protein expression in the gland and cement was determined on absolute protein abundance using an intensity-based absolute quantification (iBAQ) score calculated by MaxQuant (Table S5 and Table S6, respectively). The most expressed proteins in the gland were associated with muscle and cytoskeleton motility (Figure 3a). The majority corresponded to actin, myosin, troponin, and tropomyosin, among other contractile and structural proteins (Figure 4a). In addition, proteins involved in “adhesion, extracellular matrix and membrane” corresponded approximately to 11% of the total expression (Figure 3a), with elastin being one of the most expressed within this functional group (Figure 4a,c). Interestingly, a group of highly expressed proteins accounting for approximately 2% of the total expression remained uncharacterized or unannotated (Figure 3a). In this sense, the relative expression of unannotated proteins was like that of “laminin” and “heat shock proteins (HSPs)”, and relatively higher than “ribosomal” proteins and “histones” (Figure 4a). Minor components related to the stress response, detoxification, immunity, protein biosynthesis, proteases, proteinase inhibitors, and chemical cues were also detected in the cement gland (Figure 3a, Figure 4a,c).

**Figure 5:**
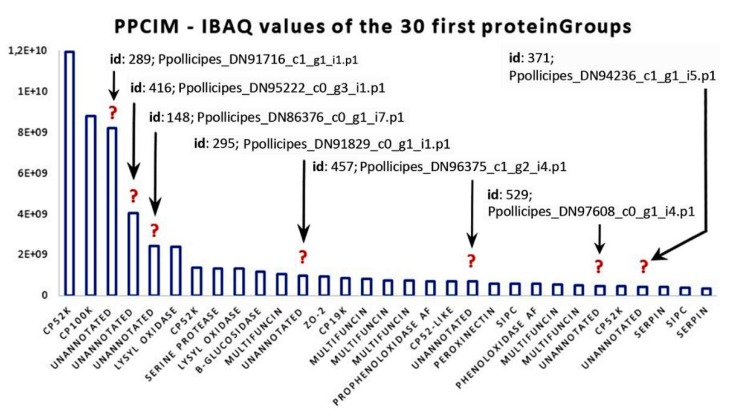
The 30 most expressed proteins in the cement proteome (PPCIM) of the barnacle *Pollicipes pollicipes*, based on the absolute protein abundance. The expression of the 30 most expressed proteins (X-axis) is represented by the intensity-based absolute quantification (iBAQ) score (Y-axis) calculated by MaxQuant. Question marks indicate those highly expressed proteins with no homology (named as unannotated in X-axis) found in the cement proteome, their proteinGroup id, and the name of the corresponding leading protein.

**Figure 6:**
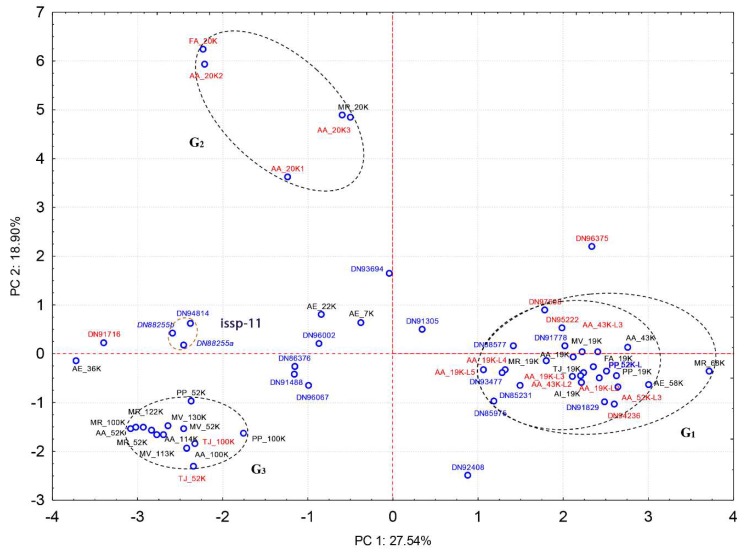
Principal component analysis based on the amino acids’ relative composition of proteins, considering previously characterized cement proteins and those proteins found during the present study, which was not possible to annotate through BLAST or conserved domains. The proteinGroups previously defined, corresponding to surface couple proteins CP19k, −43k, −58k, and −68k (G1) and CP20k (G2), and bulk proteins CP52k and −100k [19]. No unannotated proteins were found close to previously defined groups G2 and G3, but many were leaned to those of G1. The two principal components (PC) extracted explain 45.77% of the total variability of data. Blue – complete unannotated sequences; Red – incomplete unannotated sequences or incomplete cement proteins previously characterized; black – cement proteins (CPs) previously characterized.

The canonical barnacle’s cement proteins were not detected in the quantitative analyses of the cement gland at the proteomic level, except for CP100k (Figure 4a). On the contrary, the cement proteome was dominated by barnacle’s cement canonical proteins, unannotated proteins, chemical cues, and protease inhibitors (Figure 3b). Among the canonical proteins, the bulk proteins CP52k and CP100k were the most expressed, whilst among the surface-coupling CPs, only CP19k was detected at the proteomic level. Unannotated proteins were also found to be highly expressed, accounting for 23.77 % of the relative abundance (Figure 3b). Moreover, 7 proteinGroups classified as unannotated were listed among the 30 most expressed proteins (Figure 5). Noteworthy, some of those abundant unannotated proteins, such as “Ppollicipes_DN91829_c0_g1_i1.p1”, found in the cement proteome in high quantity (at position 12 in Figure 5), were also found among the 50 most expressed proteins in the gland proteome (Table S5). A new cement protein was identified (DN93583_C0_g1_i1.p2) by homology to the protein PP52k (AQA26375.1) of *P. pollicipes* [19] and automatically annotated in the proteome as CP52k like, the expression rate of which was also among the 30 most expressed proteins (at position 19 in Figure 5). According to the PCA analysis performed on the amino acid composition of this protein, it is not a bulk protein since it clusters with G1 proteins (Figure 6), being otherwise a surface couple protein. This protein corresponded to the transcript GGJN01121414.1 of the biosample SAMN08662077, bioproject PRJNA437397. The other cement proteins detected, CP100k, CP52k, and CP19k, were homologous to those previously found, ATB53757.1, ATB53756.1, and ATB53755.1, respectively [19].

In addition, other abundant proteins in the cement proteome were associated with “chemical cues” (Figure 3b). MULTIFUNCin and SIPC were the most represented (Figure 4b, Figure 5), accounting for 12.1% of the total intensity, 8.9% and 3.2%, respectively (Figure 3b). Besides, proteins involved in “adhesion, matrix, and membrane” and “protease inhibitors” were also relatively abundant, with approximately 10% and 5% of the relative abundance, respectively (Figure 3b). Some enzymes, such as lysyl oxidase, beta-glucosidase, prophenoloxidase, and phenoloxidase, were also highly expressed (Figure 4b). Minor components found in the quantitative analyses were related to catalytic activity, “cuticle”, “immunity and defense”, “muscle and cytoskeleton motility”, and “protein biosynthesis and modification”, in this order (Figure 3b, Figure 4b,c).

#### Unannotated Proteins from the Cement Proteome

Proteins without annotation, uncharacterized, or just predicted were found to be abundant in the cement proteome (Figure 3b, Figure 4b, Figure 5). Some of those proteins were also found in the gland at the proteomic (Figure 3a, Figure 4a, Table S5) and transcriptomic level (Table S9). In order to figure out the biological function of such proteins, some additional analyses were performed. A total of 32 proteinGroups were blasted against the non-redundant protein database (nr at NCBI) using automatic adjustment of the BLASTp program. Of these, a total of 17 proteinGroups remained unannotated, without any protein homology description or known conserved domains (Table S10). The results of these analyses were also included in the figures previously shown, and detailed information of the Blast search and protein sequences can be found in Table S10.

Afterwards, unannotated and uncharacterized proteins were subjected to principal component analyses (PCA) to cluster with known cement adhesive proteins (Figure 6). PCA was employed to compare the relative residue composition (%) of 21 barnacle-specific cement proteins, obtained in the present study from the *P. pollicipes* cement proteome, to 38 previously identified, classified, and characterized cement proteins of various acorn barnacle species, gathered from NCBI and the literature, belonging to 8 different barnacle species (*P. pollicipes*, *Amphibalanus amphitrite*, *A. improvisus*, *A. eburneus*, *Fistulobalanus albicostatum*, *Megabalanus rosa*, *M. volcano,* and *Tetraclita japonica*). The analysis allowed observation of the clustering patterns of the unannotated proteins with the groups of proteins previously defined [19] (Figure 6).

The two first principal components (PC1 and PC2) extracted by the PCA explained 46.4 % of the total data variation (27.54 % and 18.90 %, respectively), allowing observation of the proteins’ grouping as a function of the relative amino acid composition (Figure 6). PC1 discriminated G1 from the other two groups, while PC2 allowed for the separation of CP20k (G2) from the other two groups (G1 and G3). The three CPs from *A. eburneus*, CP36k, −22k, and −7k, identified by Naldrett and Kaplan [20], did not group with any of the three main CP groups and are putative new cement proteins, similar to the 11 unannotated cement proteins that did not group with G1, G2, or G3. Whether these cement proteins have an adhesive function or other function in the cement multicomplex is still to be determined.

The PCA situated nine of the unannotated proteins (PP95222, PP91829, PP97608, PP94236, PP93477, PP85975, PP85231, PP88577, and PP91778) in the proximity of G1 (surface-coupling proteins of CP19k, −43k, −58k, and −68k families of cement proteins), but in none of the cases in the proximity of the two other groups, G2 (surface coupling proteins of CP20k family) or G3 (bulk proteins of the families CP52k and −100k). Of the remaining 11 proteins, one of them, the most expressed of all (PP91716), was situated close to AE36k; 3 little expressed proteins (PP96002, PP93694, and PP91305) were located close to AE7k and AE22k; and the remaining 7 were found scattered among the three groups (G1, G2, and G3). One of the cement proteins picked from NCBI, AA52-3L, was misclassified according to the PCA analysis performed. Based on the relative amino acid composition of this *A. amphitrite* protein, PCA situates it in G1 (Figure 6), but according to the authors, it is a bulk protein, CP52k like [14]. In the case of being a bulk protein, it should group with G3 proteins instead of G1. The same situation was observed with the PP52k-L identified and annotated in the present work. These two proteins are smaller in length than CP52k proteins, and their physico-chemical properties also corroborate that they are surface-coupling proteins of G1 (Table 1, Table S11, Table S13).

Regarding the other characteristics of cement proteins, these are presented in Table S11 and Table 1, with the former presenting the characteristics of the 33 previously characterized adhesive proteins of various barnacle species, whose sequences were available at NCBI, and the second, the characteristics of 22 proteins found in the *P. pollicipes* cement proteome that could not be annotated by homology nor a conserved domain found, and one new CP that was automatically annotated as being a CP52k-L. Among the latter, the 10 proteins that clustered with G1 surface-coupling proteins were found to be highly disordered (> 65% disorder), presented a great percentage of its structure in the form of loops (> 60%), and a very small percentage in the form of α-helices (< 10%); more than 60% of their residues were exposed, less than 5 % were intermediate residues, and less than 35% were buried; these characteristics together with the high protein disorder estimated, agreed with the G1 protein characteristics. Their isoelectric point, aromatic index, and the aliphatic percentage also fall in the range of those of G1 proteins. 

## 3. Discussion 

### 3.1. The Proteome Profile of Pollicipes pollicipes Cement Gland

The quantitative analysis of the cement gland proteome of *Pollicipes pollicipes* was mainly composed of structural proteins and proteins involved in muscle contraction (Figure 3a), such as myosin, troponin, tropomyosin, twitchin, and titin (Figure 4a). This result is not unexpected since the cement gland’s adhesive-secreting cells form agglomerates, rather than being organized in an encapsulated gland, which makes it difficult to extract and isolate this organ. The cement gland of *P. pollicipes* was placed in the central core of the body, embedded in an apical layer of connective tissue, and surrounded by a thick layer of muscular tissue [15]. The results were consistent with previous proteomic studies in juveniles of the barnacle *A. amphitrite*, where structural proteins like titin, and proteins involved in muscle contraction were found to be significantly upregulated [25].

Besides, other elements related to the extracellular matrix, adhesion, and membranes were relatively abundant (Figure 3a). Proteins involved in such functions are expected to be found, as well as ribosomal proteins. Except for the high expression of elastin, these results are similar to previous proteomic studies made by Chen et al. [25], with structural, transcription-translation, energy, and metabolism proteins being the most significant pathway reported [25]. Specifically, two proteinGroups classified as elastin were listed within the 30 most expressed proteinGroups (Table S5). These proteinGroups with ids 1484 and 1483 (Table S7), led by the proteins Ppollicipes_DN89122_c0_g2_i1.p2 and Ppollicipes_DN96498_c0_g2_i2.p1, respectively, were found in positions 10 and 14 of the absolute abundance score ranking (see Table S5 for detailed information of the iBAQ-score). Elastin plays an important role in the extracellular matrix, conferring elasticity to the connective tissue [26], and the cement gland of *P. pollicipes* is in fact embedded in a layer of connective tissue [15]. It is noteworthy that both proteinGroups were also found in the cement proteome, with the proteinGroup 1483 being found in the three replicates (Figure 2b, Table S6), while the proteinGroup 1484 was detected in only two of the three replicates (Figure 2a, Table S4).

In addition to its fundamental role of providing elasticity to the connective tissue, elastin could be accumulated in the gland and then secreted to the base of the animal peduncle (Figure 1). Elastin can confer flexibility to the adhesive joint of the barnacle, helping the animal to return to its original position after a mechanical stimulus of the body. Elastin is insoluble, and rich in hydrophobic amino acids, such as glycine and proline, which form mobile hydrophobic regions bounded by crosslinks between lysine residues [26]. Thus, elastin has been associated with a cross-linking mechanism of bulk proteins in the cement [10].

Another protein relatively abundant in the cement gland belongs to the heat shock proteins (HSP) family. Similar to our findings, three proteins of the HSP family were detected as significantly upregulated in the proteomic profile of juveniles from the barnacles *A. amphitrite* [25]. This family was initially associated with stress-responsive pathways but then was ascribed to other functions like the regulation of developmental stages [25]. Indeed, this protein family has shown diverse roles, such as molecular chaperones, related to protein folding, assembly, and transport [27,28]. HSPs in the cement gland may be mainly involved in morphogenesis [29] and metamorphosis [30,31,32], since the samples here analyzed were obtained from juveniles of *P. pollicipes*.

On the other hand, the absence of barnacle-specific cement proteins in the gland proteome, except for the CP100k, could be associated with the complexity related to handling this type of sample, affecting the detection of those proteins at the proteomic level. Factors related to concentration, precipitation, protein digestion, and dissolution compared to the major components of the samples (muscle proteins) could affect the absolute abundances. In this respect, the total number of shared proteins between the gland and cement proteome was relatively low. Note that around 50% of the total proteins identified in the cement proteome could be found in the gland proteome (Figure 2a), while approximately 43% of the proteins found in the three replicates in the cement proteome were also found in the three replicates of the gland proteome (Figure 2b). Moreover, 790 proteins were uniquely detected in the cement proteome, representing more than the number of shared proteins with the gland (Figure 2a). Although, some works suggest that once the barnacles are established and fixed to the substrate, the production of some cement proteins could be reduced or modulated after barnacle settlement [33].

Nonetheless, CP100k was represented in the gland proteome by a cluster of six proteins from the proteinGroup id 1217 (Table S7). The leading protein of the aforementioned proteinGroup “Ppollicipes_DN94611_c0_g2_i6”, a homologue to the CP100k with accession ATB53757.1 (Protein database, NCBI), was also found with a low relative expression at the transcriptomic level (RPKM = 6.8; relative expression position 7177 in Table S9). However, the other leading protein, “Ppollicipes_DN94611_c0_g2_i4”, was found to be highly expressed at the transcriptomic level with an RPKM value of 1552.55, at a relative expression position 57 (Table S9). The CP100k above-mentioned was found highly expressed in the cement proteome, clustered in the same proteinGroup with id 241 (Table S8). Moreover, some cement proteins, such as CP52k, “Ppollicipes_DN90331_c0_g4_i4”, highly expressed in the cement proteome, were also found to be highly expressed at the transcriptomic level (RPKM = 4029.9; relative expression position 18 in Table S9), as well as the “Ppollicipes_DN91238_c1_g1_i9” (RPKM value of 1170.1; at a relative expression position 74 in Table S9). The production of other adhesive proteins is not discarded, since some unannotated proteins like “Ppollicipes_DN91829_c0_g1_i1.p1” highly expressed in the gland (Table S5), were also found in the cement proteome (at position 12 in Figure 5).

### 3.2. Unraveling the Pollicipes pollicipes Cement Proteome

The major components of the cement proteome corresponded to the barnacle’s specific cement proteins, unannotated proteins, chemical cues, adhesion proteins of the extracellular matrix (ECM) and membranes, protease inhibitors, and proteases (Figure 3b). The barnacle-specific cement proteins were the most abundant family of proteins in the cement, accounting for more than one-third (36.1 %) of the whole cement proteome (Figure 3b). Bulk proteins are hydrophobic proteins that play a fundamental role in cement self-assembly and curing, forming a framework that gives internal cohesion to the adhesive joint, provides mechanical strength, and ties up other cement proteins [13,34]. The previously reported major abundance of bulk proteins in the cement [17,35] was corroborated by the present results, where bulk proteins together were the most abundant in *P. pollicipes* cement (96.2%), with CP52k being the most abundant of all at 60.3 % (Figure 4b). The other class of adhesive proteins present in the cement, in minor amounts, was the hydrophilic surface-coupling proteins, whose function is thought to be the displacement of the water layer of the surface where adhesion will occur, and the priming and connecting of the bulk cement [13,36]. In the *P. pollicipes* cement proteome, only the CP19k surface-coupling protein was present, and at a very low amount, 3.6% of the total canonical cement proteins, with the absence of other G1 canonical proteins, namely CP20k, CP43k, and CP68k, being noteworthy. We suppose that in this species, the role that those “missing” proteins should play might be performed by some of the highly expressed unannotated proteins found (Figure 3b, Figure 4b, and Figure 5). The presence of the unannotated proteins and their putative role as adhesive proteins will be discussed further below. Surface-coupling proteins of G2 (CP20k) and CP68k of G1 (Figure 6) were not found in the *P. pollicipes* cement proteome, with their absence in accordance with the function they have been given in the cement. These proteins have been described only in calcified-base barnacles, at the interface between cement and the calcareous base [13,37,38], a structure that pedunculate barnacles do not have. So far, CP20k has never been described in membranous-base barnacles, either pedunculate or membrane-base acorn barnacles, such as *T. japonica* [39].

*P. pollicipes* cement presented a highly varied composition of other proteins than the canonical barnacle-specific cement proteins, being the second most abundant protein family of the cement proteome (12 %), composed of molecules that function as chemical cues, the MULTIFUNCin, and the settlement-inducing protein complex (SIPCs). SIPC is a glycoproteic contact pheromone that triggers conspecific larvae to induce gregarious settlement, first isolated in *A. amphitrite* [40]. It is expressed in the cuticle of larvae, and cuticle and shells of adults, and is possibly secreted in the epidermal cells that produce these tissues [41,42]. SIPC binds tightly to shell components, and also functions in biomineralization [43]. In adults, it is highly expressed in all tissues that are in contact with the cuticle, and in the shells. In the cyprids, it is found in the “footprints”, where it functions as a temporary adhesive to the explored substrates [44], simultaneously increasing the probability of other cyprids settling there in the future [45]. SIPC was present in high relative amounts in the *P. pollicipes* cement proteome. Its function there is not clear; it may function as an adhesive protein in adults as well, or it may have a pheromone function, like in barnacle cyprids, directing the conspecific larvae path along the peduncle to the substrate. In pedunculate barnacles, cyprid settle in adults’ capitulum shells or peduncle’s cuticle, walking down to the peduncle’s base in a couple of weeks [46,47]. The presence of SIPC was previously reported in adults’ acorn barnacle cement [10,14,48], but quantitatively, it was not perceived that it is so abundant there, representing 3.2% of the total *P. pollicipes* cement proteins, indicating that it may play other roles in addition to being a chemical cue, possibly as adhesive, similarly to the cyprids. In this species, the SIPC’s adhesive role may be related to the binding to chitin, rather than to minerals of the shell and biomineralization as previously proposed for acorn barnacles [43], since in pedunculate barnacles, shells are in the capitulum and do not contact with the cement. Contrarily, *P. pollicipes*’s chitinous cuticle tends to be thicker and prominent at the peduncular base, and SIPC may be one of the proteins that adhere to it. The origin of the SIPC found in the cement may be the gland, since it was also found there, although in a much smaller relative amount as compared to the cement.

*A. amphitrite* SIPC shows up to 31% similarity to α2-macroglobulins (A2M), a broad-spectrum protease inhibitor also involved in the immune response in invertebrates [49], thought to be an SIPC precursor, which from it may have evolved by duplication from an ancestral A2M gene [45,50]. Various proteinGroups of A2Μ could only be found in the gland proteome. SIPC seems to be the only protein identified so far in cyprid cement [44]. If it is the only one, or one of the few “footprint” proteins, it makes sense for it to be a protease inhibitor simultaneously to its adhesive and pheromone role, to extend “footprints” integrity, in a microorganism and protease-rich environment, such as marine benthic or intertidal ecosystems, avoiding pheromone and aggregating signal precocious termination. Indeed, the serine protease-based antifouling—Alcalase®—mode of action is the reduction of adhesiveness and removal of cyprids’ “footprints” [51].

MULTIFUNCin is a multifunctional glycoprotein cue that induces habitat selection by, and predation on, barnacles [52]. Its expression is described to be limited to exposed surfaces of epidermal origin, such as cuticle, feeding appendages, and new shell material [52], and it was found previously in another barnacle’s cement [10]. *Balanus glandula* MULTIFUNCin shares 78% nucleotide sequence homology with *A. amphitrite’s* SIPC, and it was proposed that the two proteins have a common evolutionary origin [52]. MULTIFUNCin was found in both the *P. pollicipes* gland and cement proteomes, but in a much greater relative proportion in the cement, where it makes up nearly 9% of the whole cement proteome, three times more than SIPC. Its function there may have to do with settlement induction and, based on the combined evidence, possibly with adhesion and defense, similarly to SIPC, as they both evolved from A2M. The defense role of MULTIFUNCin in the cement may be against epibionts, as was proposed for *B. glandula* [52]. MULTIFUNCin is a product of epidermal origin [52], and the cement gland also originates on epidermal cells [53], so it is possible that MULTIFUNCin, as well as SIPC, are both produced in the cement gland. Interestingly, both MULTIFUNCin and SIPC showed high isoform diversity, with more than 20 and 12 proteinGroups for those proteins found, respectively (Table S8). The different proteinGroups are supported by the presence of unique peptides, suggesting that these isoforms of the genes have evolved to play different roles in the cement secretion, e.g., interact specifically with a certain species. In this sense, evolutionary analyses must be conducted to understand the diversity of these protein families and their functional role in barnacle cement.

Apart from A2M, which was only found at the cement gland in a minute relative abundance, a good amount (4.5%) of serine protease inhibitors (SERPINs) and other protease inhibitors, such as pacifastin, were found in the cement. Protease inhibitors’ presence in the cement makes a lot of sense, since the proteases of other species, namely micro- and macrofoulers, are a threat to cement integrity. Serine proteases and other proteases, including metalloproteinases and disintegrin, were found in *P. pollicipes* cement in a relative amount of 2%, in accordance with the previous findings in *Chthamalus fragilis* [54]. Evidence of a prophenoloxidase system, including activating factors, serine proteases, and SERPINs, were described in barnacle’s cement [48]. This proteolytic cascade is highly conserved in crustaceans, participating, i.e., in cuticle sclerotization via enzymatic oxidation and antimicrobial activity [55,56]. The lipopolysaccharide and β-1,3-glucan-binding protein (LGBP), which is demonstrated to activate the prophenoloxidase cascade in insects, shrimps, and crabs [57], was also present in the *P. pollicipes* cement.

Other oxidases, such as lysyl oxidase (5.5%) and other peroxidases, namely peroxinectin (1%), were present in the *P. pollicipes* cement at high relative concentrations. These enzymes are responsible for performing post-translational modifications of cement proteins. Peroxidases are involved in oxidizing phenolic cross-linking chemistry, and lysyl oxidase is involved in lysine/arginine protein cross-linking, reactions also observed in collagen’s and elastin fibrils’ cross-linking [10]. Its presence is in accordance with previous findings, where their role was assigned to the modification of cement components, such as CP43k [10]. A peroxinectin characterized in *A. amphitirite* prefers phenolic chemistry over amino acids [10]. Similarly, peroxinectin and peroxidases have been reported as cross-linkers in insect adhesives and eggshells [58,59,60].

#### Unannotated Proteins

A total of 17 unannotated proteins accounted for around 24% of the cement proteome. Some of these proteins were also found to be highly expressed in the gland proteome, as well as in the gland transcriptome. Although CP19k was found among the annotated proteins, the presence of surface-coupling protein was relatively low, thus some unannotated proteins may be playing this function in the interface of bulk proteins and the substrate. As previously described, surface-coupling proteins are structurally disordered, which confers them elasticity [61,62,63,64] and drives self-assembly into nanofibers and mesh structures, similar to amyloid-like fibril aggregations that were also found in bulk proteins CP100k and CP52k [34,35,65,66,67,68]. The properties of the CPs in the barnacle adhesive multicomplex is thought to rely on the specific sequence of a bias amino acid composition, with adequate properties for the function each one delivers on the composite, featuring different pI, hydrophobicity, charge, etc., and often presenting alternate blocks of repetitive sequences, or not [14,17,19,69,70].

Among them, the PCA and the analysis of the physical and chemical parameters revealed that at least 9 of the 17 unannotated proteins showed most of the requirements to be considered new adhesive proteins. These new cement proteins are highlighted in gray in Table 1. Noteworthy, in Figure 6, these proteins shown with the codes PP95222, PP91829, PP97608, PP94236, PP93477, PP85975, PP85231, PP88577, and PP91778 are placed nearby those of G1, the group of surface-coupling proteins of the families CP19k, −43k, −58k, and −68k, but in none of the cases in the proximity of the two other groups, G2 (surface coupling proteins of CP20k family) or G3 (bulk proteins of the families CP52k and −100k), based on their amino acid composition. They were found to be disordered (>65% disorder), with a great percentage of its structure in the form of loops (>60%), on the contrary to the percentage in the form of α-helices (<10%), which are surface-coupling protein characteristics. Besides, their amino acid residues were exposed (>60%), less than 5% were intermediate residues, and less than 35% were buried. Their isoelectric point, aromatic index, and the aliphatic percentage also fall in the range of those of G1 proteins. Additional information on these proteins can be found in Table S12.

The secondary structure predicted for the protein AA_52k-L3 of *A. amphitrite* [14] deposited at NCBI is not in accordance with that of bulk proteins CP52k or CP100k. It is a 100% disordered protein, totally composed of loops, with 90% of the residues exposed. These characteristics match with the G1 surface-coupling proteins, as also determined by PCA. Additionally, the secondary structure characteristics predicted for the *T. japonica* TJ52k, classified in bulk proteins by PCA, do not match those of the cement protein group (G3). The degree of disorder is too high for a bulk protein, as well as the percentage of loops, and the reduced value of exposed residues. This may occur because the sequence of the protein analyses is incomplete [39] and too small as compared to the total length expected, which does not all the unveiling of the true secondary structure of this supposed bulk protein.

Concerning other unannotated proteins not in the vicinity of G1, they are cement proteins that may be adhesive or not. If they are adhesive proteins, they belong to different adhesive protein families with a different amino acid composition and physico-chemical characteristics. Otherwise, they are new cement proteins but with an unknown function. Three of these proteins (DN94814, DN88255a, DN88255b) grouped together between G2 and G3 (Figure 6) and were found to have conserved domains similar to those of a *Sacculina yatsui* internal-specific secretory protein (issp-11), a member of hemolymph juvenile hormone-binding protein (JHBP) superfamily.

## 4. Materials and Methods

### 4.1. Sampling, Protein Solubilization, and Extraction

Six immature *Pollicipes pollicipes* (Crustacea: Cirripedia: Pedunculata) specimens (<12 mm in rostro-carinal length) were collected on the rocky shore of Praia da Memória beach, Porto, Portugal, during low spring tide in August 2017. Individuals with undeveloped ovaries were selected owing to the proximity of the ovary to the cement gland to avoid contamination. Animals were brushed to clean epibionts, transported to the laboratory on ice, further swept with an ethanol-soaked cellulose cloth, and dissected upon arrival. The cement gland was located according to previous studies [15]. The tissues collected were kept frozen at −80 °C until protein homogenization and extraction, in sodium dithionite (SDT) buffer (2% SDS, 100 mM Tris/HCl pH 7.6, 0.1 M DTT) according to Campos et al. [71]. Tissues were first homogenized using ultrasounds (Vibra Cell, Sonics and Materials Inc., Newtown, CT, USA) at 60 Hz intensity, then mechanically disrupted using microbeads (Precelly’s, Bertin instruments, Montigny-Le-Bretonneux, France), followed by incubation in SDT for 14 h with agitation (450 rpm) in a thermomixer at room temperature. Samples were then centrifuged at 16,000 g for 20 min, the supernatant collected, the protein concentration determined by spectrophotometry (Synergy HT, BioTek Instruments, Winooski, VA, USA) at 280 nm, and stored at −80 °C until further analysis.

### 4.2. LC-MS/MS Analyses

Provided lysates were incubated for 30 min with 5 mM tris(2-carboxyethyl)phosphine (TCEP) at 56 °C. The solution was brought to 10 mM TCEP and 10 mM methyl methanethiosulfonate (MMTS) for 15 min to reduce and protect cysteine residues, respectively. Protein purification, protein digestion, and peptide purification were performed according to a slightly adapted single-pot solid-phase-enhanced sample preparation (SP3) protocol [72,73]. Sequencing-grade trypsin (Promega, Fitchburg, WI, USA) was added at a ratio of 1:50 weight per weight in 50 mM HEPES, pH 8. After overnight incubation at 37 °C, beads containing the digested peptides were slightly acidified using 10% formic acid (FA), and shaken and incubated overnight at room temperature, after raising the acetonitrile concentration to at least 95%. Adsorbed peptides were washed once with pure acetonitrile (ACN) and then air dried. Peptides were eluted in the first step with 20 µL 2% dimethyl sulfoxide (DMSO) for 30 min and in the second step with 20 µL 0.065% FA, 500 mM KCl in 30% acetonitrile for 30 min. Peptides were vacuum dried and dissolved in 0.2% trifluoroacetic acid/3% ACN for subsequent ultracentrifugation (50,000× *g*, 30 min, RT). LC-MS/MS analyses of purified and desalted peptides were performed on a Dionex UltiMate 3000 n-RSLC system, connected to an Orbitrap Fusion^TM^ Tribrid^TM^ mass spectrometer (Thermo Scientific, Waltham, MA, USA). Peptides of each sample were loaded onto a C18 precolumn (3 μm RP18 beads, Acclaim, 0.075 mm × 20 mm), washed for 3 min at a flow rate of 6 µL/min, and separated on a C18 analytical column (3 mm, Acclaim PepMap RSLC, 0.075 mm × 50 cm, Dionex, Sunnyvale, CA, USA) at a flow rate of 200 nL/min via a linear 120 min gradient from 97% MS buffer A (0.1% FA) to 25% MS buffer B (0.1% FA, 80% ACN), followed by a 30 min gradient from 25% MS buffer B to 62% MS buffer B. The LC system was operated with the Chromeleon software (version 6.8, Dionex) embedded in the Xcalibur software suite (version 3.0.63, Thermo Scientific). The effluent was electro-sprayed by a stainless-steel emitter (Thermo Scientific). Using the Xcalibur software, the mass spectrometer was controlled and operated in the “top speed” mode, allowing the automatic selection of as many doubly and triply charged peptides in a 3-s time window as possible, and the subsequent fragmentation of these peptides. Peptide fragmentation was carried out using the higher energy collisional dissociation mode and peptides were measured in the ion trap (HCD/IT).

### 4.3. Protein Identification and Quantitative Proteomic Analyses

MS/MS raw data files comprising three biological replicates per sample studied, gland (DATASET S1) and cement (DATASET S2), were processed independently against a *P. pollicipes* custom proteins database using MaxQuant freeware (version 1.6.2.3) [74]. The protein database obtained with TransDecoder (version 5.5.0) comprised 65,291 coding sequences (DATASET S3) from the *P. pollicipes* transcriptome. From these, 39,123 (60% of the total) were annotated using a local BLAST with BLASTp program, against the non-redundant protein database (nr database: ftp://ftp.ncbi.nlm.nih.gov/blast/db; accessed November 1 2018), setting a cut-off e-value of 1e^−3^. The DATASETs S1, S2, and S3 were deposited at the Mendeley Data repository with the following Digital Object Identifiers: http://dx.doi.org/10.17632/bc65592ymt.1, http://dx.doi.org/10.17632/pgkf3mtb4m.1, and http://dx.doi.org/10.17632/92wsg4g3mt.1, respectively. It is noteworthy that this transcriptome was previously published [75] and was obtained from the same specimens used to profile this proteome. The transcriptome is publicly available at Transcriptome Shotgun Assembly (TSA, NCBI), deposited under accession GGJM01000000.

MaxQuant parameters for protein identification were: MS and MS/MS tolerances of 20 ppm and 0.5 Da, respectively; two missed tryptic cleavages were allowed; PSMs were accepted at a 1 % false discovery rate (FDR); and trypsin was selected for protein cleavage. The modification of cysteine by MMTS (methylthiolation) was set as a fixed modification, while oxidation of methionine and acetylation of protein N-terminus were chosen as variable modifications. Protein quantification was based on the approximate absolute protein abundance an intensity-based absolute quantification (iBAQ) score calculated by MaxQuant. Venn diagrams were used to identify the shared proteins among the majority proteins of each replicate and figures were built using an online free tool, available at the webserver of the Bioinformatics and Evolutionary Genomics Center (BEG/Van de Peer Lab site, Ghent University, Belgium, http://bioinformatics.psb.ugent.be/webtools/Venn/).

### 4.4. Data Filtration and Downstream Analyses 

Downstream analyses as data filtration of proteinGroups obtained with MaxQuant were performed using Perseus freeware (version 1.6.2.3) [76]. Original data filtration included contaminants and REV_ removal, as well as those proteins only identified by site. Afterwards, the absolute intensity (iBAQ) of filtered proteinGroups was log(x)-transformed and only those proteinGroups with 3 valid values (of 3 possible) per row were considered. For the protein expression analyses, only those proteins found in the three replicates per sample were considered. The resulting matrix containing all filtered proteinGroups was exported and manually reviewed using a set of keywords regarding the family of proteins found in barnacle cement or related organisms.

### 4.5. Characterization of Unannotated Cement Proteins

Protein sequences found in the cement proteome without hit or annotations were blasted online against the non-redundant protein database (nr at NCBI), using automatic adjustment of the BLASTp program. Afterwards, proteins sequences were re-annotated according to the hit description or considering conserved domains. In case proteins remained without a hit or lacked a conserved domain, as previously described for most cement proteins, they were characterized using the ProtParam tool from EXPASY (http://web.expasy.org/protparam/), including molecular weight and isoelectric point, instability index, hydropathy, percentage of positive, negative and aromatic residues, and aliphatic index. Predictions on the secondary structure composition, solvent accessibility, and protein disorder were performed by PredictProtein [77] (https://www.predictprotein.org/) from protein sequences by Meta-Disorder [24]. It is a system of neural networks that combines several original prediction methods with the evolutionary profiles and sequence features that correlate with the protein disorder, such as the predicted solvent accessibility and protein flexibility (loops), which were used. Principal component analysis (PCA) was used to analyze the composition of the residue (%) of 21 barnacle-specific cement proteins (unannotated proteins) obtained in the present study, in comparison to 38 cement proteins of various acorn barnacle species (order Sessilia), and three proteins (CP100k, −52k, and −19k) of *P. pollicipes* (order Scalpelliform) deposited at NCBI and the literature. Only 20 amino acids were considered, for aspartic acid and asparagine were analyzed together, as well as glutamic acid and glutamine, since in some cases, CPs’ data delivered by the authors was in this form (one value for each of these two pairs of amino acids). Statistica (version 8.0, StatSoft, Inc.) was used to perform the PCA analyses.

## 5. Conclusions

This work revealed for the first time the whole proteome of the adhesion system of the barnacle *P. pollicipes*. The gland proteome was found to be dominated by proteins involved in muscle, cytoskeleton, and some uncharacterized proteins, while minor components are involved in the stress response, detoxification, immunity, protein biosynthesis, protease inhibitors, and chemical cues. On the contrary, the cement is mainly composed of the barnacle’s adhesive proteins, unannotated proteins, enzymes, chemical cues, and protease inhibitors. The bulk proteins accounted for one-third of the cement proteome, with CP52k being the most abundant. Nonetheless, some of the most expressed proteins found lacked homology to any known protein. However, some of these unannotated proteins were found to be highly expressed both in the gland and the cement proteome, as well as at the transcriptomic level. Considering the lack of homology with known proteins, their amino acid composition, molecular weight, isoelectric point, and other chemical physical properties, we conclude that nine of them are surface-coupling adhesive proteins of G1, related to CP19k, as revealed by principal component analyses. Moreover, the low relative expression of known surface coupling indicates that those unannotated proteins could be playing a fundamental role in the bulk proteins–substrate interface. The other 12 unannotated proteins found in the cement may be adhesive or not, but their characteristics fall outside those of the adhesive protein groups characterized so far in the literature. More analyses must be performed in the future to validate the role of the unannotated proteins. The thorough quantitative description of the proteins analyzed, both in the gland and cement from the barnacle *P. pollicipes*, provides clues to understand the diversity of adhesive proteins and their function in cirripeds, serving as complementary information to update the barnacles’ cement adhesion model.

## Figures and Tables

**Table 1:** Characteristics of the 21 unannotated proteins identified in *Pollicipes pollicipes* cement proteome, and of a new CP protein annotated as 52k-like (DN93583_C0_g1_i1.p2), ranked according to the intensity-based absolute quantification (iBAQ) value. No. Res – number of residues; MM – molecular mass; pI – isoelectric point; Neg. res. – negative residues (sum of Asp and Glu); Pos. res. - positive residues (sum of Arg, His and Lys);. In grey are proteins that have clustered with the adhesive proteins of group 1 (G1; surface-coupling proteins).

iBAQ Rank	Barnacle Specific Leading Cement Proteins	No. Res	MM(x1000)	pI	Instability Index ^a^	Hydropathy (GRAVY) ^b^	% Neg. res.	% Pos. res.	Aliphatic Index ^c^	Aromatic res. (%)	Secondary Structure Composition (%)			Solvent Accessibility (%)				Protein Disorder (%)
											Loop	α-Helix	β-Sheet	Exposed	Interm		Buried	
*3*	*DN91716_c1_g1_i1.p1*	*390*	*45.4*	*10.4*	*50.95*	*−0.142*	*6*	*47*	*88.69*	*17.5*	*53.13*	*25.38*	*19.49*	*26.15*		*7.44*	*66.41*	*0.51*
*4*	*DN95222_c0_g3_i1.p1*	*145*	*15.3*	*9.1*	*29.78*	*−0.768*	*19*	*22*	*64.48*	*0.7*	*63.45*	*10.34*	*26.21*	*72.41*		*3.45*	*24.14*	*100.0*
5	DN86376_c0_g1_i7.p1	398	42.8	9.57	41.03	0.260	7	11	104.95	6.8	40.7	35.18	24.12	40.20		0.00	59.80	1.78
12	DN91829_c0_g1_i1.p1	269	25.3	4.1	49.53	0.111	28	7	79.93	2.2	92.19	5.58	2.23	64.68		1.86	33.56	69.58
19	DN93583_c0_g1_i1.p2	243	23.7	5.4	34.24	−0.293	11	9	72.3	0.4	91.8	6.1	2.1	68.7		2.1	29.2	91.77
*20*	*DN96375_c1_g2_i4.p1*	*281*	*27.9*	*4.0*	*21.94*	*−0.67*	*51*	*14*	*62.17*	*0.8*	*77.94*	*6.05*	*16.01*	*54.09*		*2.85*	*43.03*	*45.20*
*26*	*DN97608_c0_g1_i4.p1*	*146*	*14.4*	*10.7*	*20.79*	*−0.738*	*9*	*21*	*52.95*	*2.1*	*92.84*	*3.42*	*2.74*	*71.92*		*2.74*	*25.34*	*100.0*
*28*	*DN94236_c1_g1_i5.p1*	*312*	*29.3*	*6.7*	*21.37*	*−0.013*	*21*	*20*	*69.01*	*2.5*	*97.76*	*0.00*	*2.24*	*90.38*		*0.32*	*9.29*	*93.59*
36	DN94814_c0_g2_i2.p1	240	26.3	10.6	24.38	0.12	12	36	101.58	8.3	50.00	43.33	6.67	37.08		7.08	55.83	4.58
38	DN93477_c2_g1_i1.p1	353	37.4	8.5	37.24	−0.532	44	46	79.52	0.6	90.93	1.42	7.65	68.56		3.40	28.05	64.58
48	DN92408_c0_g3_i2.p1	392	43.5	11.5	25.19	−0.148	13	37	89.92	4.3	96.68	0.00	3.32	94.90		0.51	4.59	96.17
57	DN85975_c0_g1_i3.p2	203	19.8	4.8	43.26	−0.159	16	11	55.91	6.9	94.58	5.42	0.00	88.67		1.48	9.85	95.07
60	DN88255_c1_g1_i1.p1	265	30.0	11.3	49.76	−0.206	14	45	96.45	6.9	43.77	41.17	9.06	50.94		7.55	41.51	7.17
79	DN85231_c0_g1_i1.p1	246	23.7	12.4	25.83	0.049	3	20	77.03	1.6	96.34	0.00	3.66	67.48		1.22	31.30	87.80
87	DN88577_c1_g1_i6.p1	105	10.3	6.8	4.04	0.25	5	5	75.24	4.5	60.00	0.00	40.00	60.95		4.76	34.29	62.86
89	DN96067_c1_g1_i3.p1	183	20.2	10.8	19.96	0.138	8	28	106.94	7.6	34.97	19.13	45.9	43.72		8.74	47.54	2.73
95	DN96002_c6_g1_i8.p1	238	26.9	10.0	57.57	−0.786	23	37	57.77	7.6	59.24	12.61	26.15	59.24		4.62	36.13	51.68
102	DN91305_c0_g1_i7.p3	190	20.5	6.9	38.75	−0.453	21	21	69.68	4.2	46.84	14.21	38.95	55.79		5.79	38.42	26.32
103	DN91778_c0_g1_i1.p1	606	59.3	5.0	55.78	−0.332	49	32	67.79	0.9	99.05	0.00	0.50	96.20		0.00	3.80	97.95
106	DN91488_c0_g1_i1.p1	204	22.3	9.0	46.8	−0.23	17	22	68.63	7.4	39.71	53.92	6.37	52.94		2.45	44.61	29.90
108	DN88255_c1_g2_i2.p1	265	29.9	10.8	54.04	−0.186	18	42	101.62	5.7	46.79	37.36	15.85	41.51		5.66	52.83	7.55
141	DN93694_c2_g1_i2.p1	302	30.9	8.6	36.49	−0.364	20	24	70.13	4.0	3.31	96.69	0	56.95		4.97	38.08	84.44

^a^ Instability index (II) - provides an estimate of the stability of the protein in a test tube, depending on the presence of certain dipeptides [21], the occurrence of which is significantly different in the unstable proteins compared with those in the stable ones. A protein whose instability index is smaller than 40 is predicted as stable, a value above 40 predicts that the protein may be unstable. ^b^ GRAVY - Grand Average of Hydropathy - The GRAVY value for a peptide or protein is calculated as the sum of hydropathy values [22] of all the amino acids, divided by the number of residues in the sequence. Values define relative hydrophobicity of amino acid residues, the more positive the value, the more hydrophobic in the amino acids located in that region of the protein. ^c^ Aliphatic index of a protein is defined as the relative volume occupied by aliphatic side chains (alanine, valine, isoleucine, and leucine). It may be regarded as a positive factor for the increase of thermostability of globular proteins [23]. Protein disorder – percentage of disordered regions as compared to the total protein sequence length predicted by Meta-Disorder [24].

## Supplementary Materials

Supplementary materials can be found at https://www.mdpi.com/1422-0067/21/7/2524/s1.
